# Advocating for PCR-RFLP as molecular tool within malaria programs in low endemic areas and low resource settings

**DOI:** 10.1371/journal.pntd.0011747

**Published:** 2023-11-08

**Authors:** Mergiory Y. Labadie-Bracho, Malti R. Adhin

**Affiliations:** 1 “Prof. Dr. Paul C. Flu” Institute for Biomedical Sciences, Kernkampweg, Paramaribo, Suriname; 2 Anton de Kom Universiteit van Suriname, Faculty of Medical Sciences, Department of Biochemistry, Kernkampweg, Paramaribo, Suriname; Charité University Medicine Berlin, GERMANY

## Abstract

The road to malaria elimination for low- and middle-income countries is paved with obstacles, including the complexity and high costs of advanced molecular methods for genomic analysis. The usefulness of PCR-RFLP as less complex and affordable molecular surveillance tool in low-endemic malaria regions was assessed in a cross-sectional study conducted in Suriname, currently striving for malaria elimination, but plagued by recent *P*. *vivax* outbreaks. Molecular analysis of two highly polymorphic genes *Pvmsp-1 F2* and Pv*msp-3α* was performed for 49 samples, collected during October 2019 through September 2021 from four different regions with varying malaria transmission risks. RFLP-profiling revealed that outbreak samples from three indigenous villages, almost exclusively, harbored a single clonal type, matching the “Palumeu” lineage previously described in 2019, despite multiple relapses and drug pressure exerted by mass drug administration events, suggesting a limited *P*. *vivax* hypnozoite reservoir in Suriname. In contrast, isolates originating from Sophie, a mining area in neighboring French Guiana displayed a highly heterogeneous parasite population consistent with its endemic malaria status, demonstrating the differentiating capacity and thus the usefulness of PCR-RFLP for *P*. *vivax* genetic diversity studies. Outbreak reconstruction emphasized the impact of undetected human movement and relapses on reintroduction and resurgence of *P*. *vivax* malaria and PCR-RFLP monitoring of circulating parasites guided the roll-out of targeted interventions. PCR-RFLP seems a suitable molecular alternative in low-endemic areas with restricted resources for outbreak analysis, for monitoring the spread or containment of circulating strains and for identification of imported cases or potential foci.

## Author summary

The global malaria landscape has changed considerably since 2000, with a drastic reduction of overall malaria cases, but a regional increase of *Plasmodium vivax* malaria. Suriname is a South American country striving for malaria elimination and *Plasmodium vivax* malaria is the main impediment, mostly due to the parasite’s ability to stay dormant in the liver and relapse within months/years after the initial infection, triggering new outbreaks.

As most low- and middle-income countries we are confronted with the lack of effective and affordable molecular tools to assess and monitor the genetic diversity of circulating *P*. *vivax* populations. We report the successful application of the PCR-RFLP technique in determining genetic signatures of *Plasmodium vivax* isolates, obtained from four different regions with varying malaria transmission risks. RFLP-profiling revealed that outbreak samples from three indigenous villages, almost exclusively, harbored a single clonal type, suggesting a limited *P*. *vivax* hypnozoite reservoir in Suriname. In contrast, isolates originating from Sophie, a mining area in neighboring French Guiana displayed a heterogeneous parasite population, consistent with its endemic malaria status. We demonstrated the usefulness of PCR-RFLP for assessments of *P*. *vivax* genetic diversity, outbreak analysis and identification of imported cases in a low-transmission setting, where resources are limited.

## Introduction

The global malaria landscape has changed considerably since 2000, with a drastic reduction of overall malaria cases, but still accounting for more than 241 million malaria cases worldwide in 2020 [1].

However, the regional incidence of *P*. *vivax* malaria increased, accentuating the need to understand the parasite population genetics.

Insight in the genetic diversity of *Plasmodium* spp. has not only been extremely valuable in the identification of malaria drug resistance associated markers, but also enables the distinction between reinfection, recrudescence and relapse [2] and even facilitates tracking of the origin of an isolate [3], which is particularly important to signal possible source(s) of outbreaks. Furthermore, global and local mapping of *P*. *vivax* population structure [4] will provide detailed knowledge of the spatial distribution, transmission and clinical burden of *P*. *vivax*, which is required to set attainable control targets and strategize about roll-out of interventions.

Consequently, epidemiological data in combination with genetic information can guide control and elimination strategies of National Malaria Programs.

Numerous approaches have been used to examine the genetic relationships between *Plasmodium* spp. and molecular methods have proven to be valuable tools for malaria outbreak investigations [5,6]. Recent advancements in genetic techniques like next-generation sequencing (NGS) and whole genome sequencing (WGS) have further increased the DNA sequencing capabilities [7], providing a far greater level of information than classic fingerprinting methods. Furthermore, WGS of malaria parasites has also been used more commonly for identification and monitoring of the status of malaria drug resistance associated markers. However, despite the obvious advantages of the newer technologies, the enormous data generated from WGS, and the complexity of the bioinformatics analysis requires strong computational expertise and sophisticated computational resources [8]. An alternative advanced technique such as high-throughput single nucleotide polymorphisms (SNPs) barcoding, a targeted sequencing technique, may offer a more feasible genotyping strategy, but still requires extensive bioinformatic analysis besides validation or development of a local barcode to accurately reflect the country’s parasite diversity [9].

Besides, the introduction of genome sequencing even into routine health care in Western countries has been slow, partly because of concerns about affordability. A review of economic evaluations of exome sequencing in 2018 for clinical practices reported cost estimates all above $500 for a single test of exome sequencing [10], on top of an initial investment for NGS equipment, which can cost well over $100K and requires an additional annual maintenance cost of ten thousands of dollars.

The hurdles encountered by most low- and middle-income countries (LMIC) extends across the borders of funding and lack of state-of-the-art equipment, as complex technical training and highly advanced skill requirements for the management and clinical interpretation of extensive sequencing data, limit operational implementation at the country level.

Therefore, the availability of effective, but less complex and affordable molecular tools is of the utmost importance for LMIC on the road to malaria elimination to successfully implement timely measures and to evaluate the effectiveness of targeted interventions.

The relative simplicity and low costs of a traditional fingerprinting method as PCR of highly polymorphic genes combined with Restriction Fragment Length Polymorphism (PCR-RFLP) may be a good option for genetic diversity assessments in resource limited settings.

PCR- RFLP can be performed in any basic molecular laboratory setting, without major capital investments for the lab set-up and within this study, the complete testing of samples for two genes using PCR-RFLP could be accomplished for a unit cost below $50.

Recent use of this tool has been successful in *Plasmodium vivax* (*P*. *vivax*) investigations, to provide insight in genetic diversity in Panama [11] and for reconstruction of a malaria outbreak in a low transmission area [12]. In addition, PCR-RFLP has been utilized to assess the geographic origin of *Plasmodium falciparum* strains *(P*. *falciparum)* in Central America [3].

*Plasmodium vivax* is currently the main impediment for malaria elimination in Suriname and we therefore set out to explore the further utilization of PCR-RFLP as molecular tool in malaria elimination settings.

Traditionally, evaluation of genetic diversity and dynamics of *P*. *vivax* parasite populations with PCR-RFLP is determined via assessment of highly polymorphic genes such as the *Pvmsp-1* and *Pvmsp-3α* genes. *Pvmsp-1 F2* codes for the merozoite surface protein 1 and encompasses a region (F2) of extensive allelic diversity [13]. *Pvmsp-3α* codes for the merozoite surface protein 3 and displays considerable sequence diversity in the central domain of the molecule with numerous point mutations and large insertions and deletions [14].

A cross-sectional survey was conducted in a two-year period from October 2019 to September 2021 on subsets of *P*. *vivax* samples, diagnosed in Suriname and originating from different regions with varying risks of malaria transmission, to assess the capabilities of PCR-RFLP as tool for low endemic malaria regions.

## Methods

### Ethics statement

Informed verbal consent for molecular malaria research was provided by all patients upon enrollment in the National Malaria Gene Bank.

Ethical clearance for the NMGB was obtained by the National Ethics Committee within the Ministry of Health (VG007-08).

### Study settings

Suriname is a small country and part of the Guiana shield, located on the northern coast of South America; notorious in malaria history for the highest annual parasite incidence and concentration of *Plasmodium falciparum* cases in the Americas in 2004. Incessant national surveillance, persistent implementation of control measures and innovative interventions resulted in a steep decline of the number of cases, from more than 10.000 cases in 2003 to 214 cases in 2019, leading Suriname to the brink of malaria elimination.

Current data on malaria incidence in Suriname is shown in Table 1 Malaria incidence data for Suriname (2019–2021) (data Ministry of Health Malaria Program Suriname [NMP]).

**Table 1:** Malaria incidence data for Suriname (2019–2021).

Year	Total Malaria cases	*Plasmodium falciparum*	*Plasmodium vivax*		
		Imported	Imported	Introduced^1^	Indigenous (%) ^2^
2019	214	13	101	N/A	100 (36.0%)
2020	244	2	95	N/A	147 (89.1%)
2021	75	5	46	2	22 (68.2%)

N/A: not available

^1^ Registration of introduced cases in the country started since October 2021

^2^ Cases from Pelele Tepoe as percentage of all indigenous *P*. *vivax* cases

Indigenous *P*. *falciparum* cases have not been registered since September 2018, while *P*. *vivax* malaria is still incidentally transmitted in the tropical rainforest of the interior, due to the vast presence of the vector (*Anopheles darlingi*) and the increased population movement of illegal mobile miners, mostly Brazilians entering the country through French Guiana and travel of villagers among communities in Suriname, Brazil, Guyana, and French Guiana.

Malaria elimination efforts are particularly challenged by the restricted access to remote communities in the interior with only boat and air transport possibilities, along with a limited healthcare infrastructure. Nevertheless, more than 90% of the total malaria cases between 2004 and 2016 were considered as imported, mostly from Brazil nationals involved in illegal gold mining activities travelling from French Guiana [15].

However, with recent *P*. *vivax* outbreaks in 2019 and 2020 in indigenous villages, previously free of malaria, the fraction of imported *P*. *vivax* cases decreased to 50.2% and 39.3% of the *P*. *vivax* cases in 2019 and 2020 respectively (data Ministry of Health Malaria Program Suriname [NMP]).

### Study design

A cross-sectional survey was designed with subsets of *P*. *vivax* samples, originating from four different regions with varying risks of malaria transmission, to gain insight into possible geographic genetic signatures.

During the two-year study period (October 2019 to September 2021), 396 single *P*. *vivax* malaria cases were diagnosed in the country, consisting of 38.1% imported and 61.9% indigenous cases, presumably originating from 36 different locations in neighboring countries and 7 locations within Suriname.

Two *P*. *vivax* outbreaks in Amerindian villages Palumeu and Pelele Tepoe generated the majority (94.3%) of the indigenous cases.

Four study regions (Palumeu, Pelele Tepoe, Apetina (Puleowime) and Sophie) were selected, based on their highest registered numbers of *P*. *vivax* samples during the study period, as a minimum number of samples for examination were required for the attempt to identify possible genetic signatures per region.

**Fig 1:**
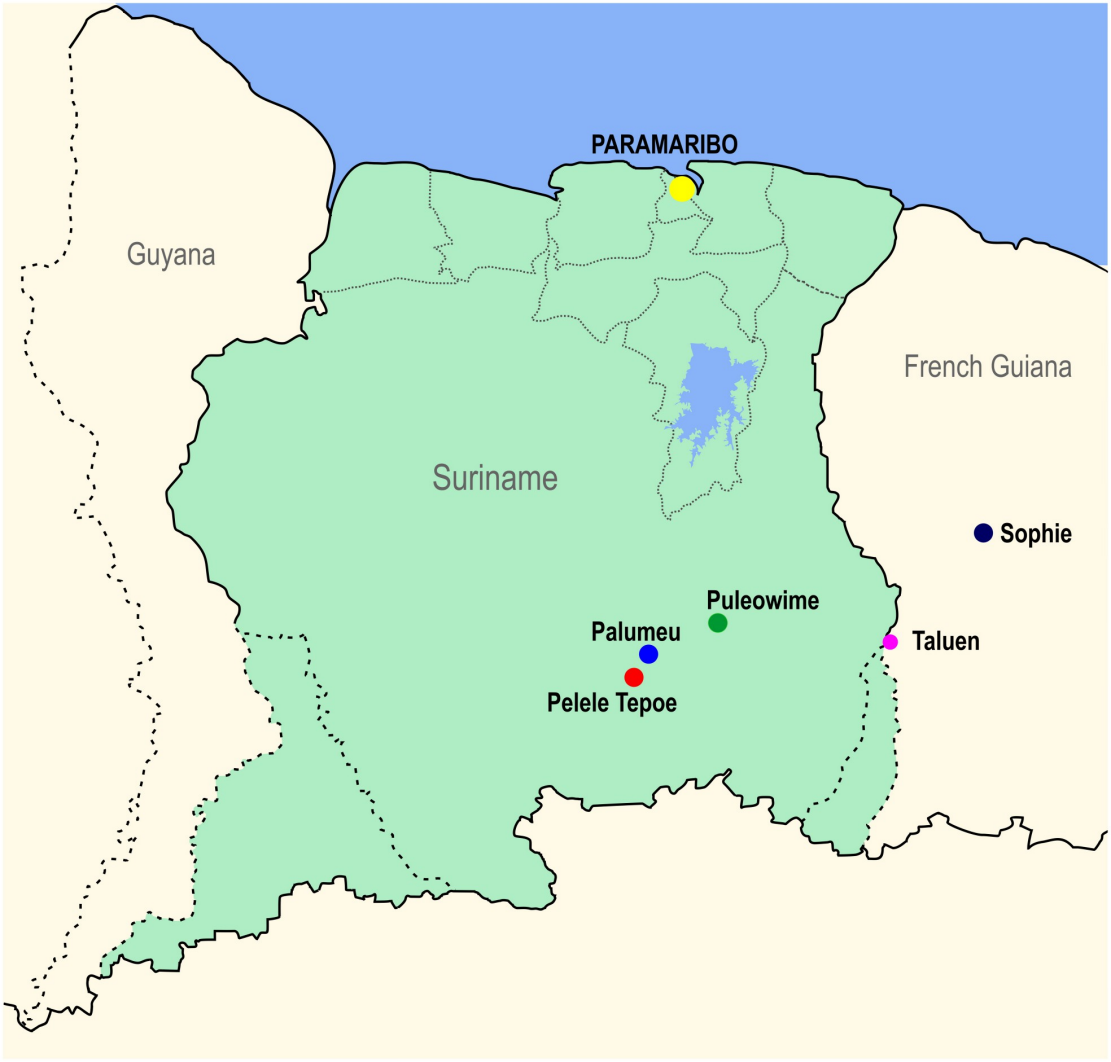
Map of Suriname with study sites. The study sites are represented by colored circles. Paramaribo, the capital of Suriname is not a study site, but is shown in the picture for reference purposes. This figure was created using a base map made with *Natural Earth*, free vector and raster map data *@ naturalearthdata*.*com in QGIS version* version 3.32.2-Lima and was retraced and edited using Corel Draw X7.

The bulk of malaria cases originated from 2 indigenous villages, namely Palumeu and Pelele Tepoe. The four study regions (Palumeu, Pelele Tepoe, Apetina (Puleowime) and Sophie) and the capital Paramaribo are pictured in Fig 1.

### Palumeu

Cases from the Palumeu outbreak have been documented from March 2019 to January 2020 (n = 58) and the index case was presumably an unregistered visitor from neighboring Brazil.

As a single clonal lineage was demonstrated in a previous study [12], only six samples of the 41 outbreak samples within the study period were included.

In addition, two other samples from Palumeu were included, diagnosed 12 months and 17 months after the outbreak, respectively.

### Pelele Tepoe

Pelele Tepoe is an Amerindian village located in the southern part of the rainforest of Suriname, with almost 500 inhabitants, which had been malaria-free for several years till the recent outbreak. The outbreak lasted from November 2019 to August 2021 with 183 confirmed cases and twenty-three (n = 23) samples were included for genetic RFLP profiling. The samples were selected throughout the outbreak, to have a fair representation of the different gaps and peaks along the timeline to enable molecular reconstruction of this outbreak. Eighty percent (80%) of participants with a travel history outside Suriname were included to spot imported cases and for participants without a cross border travel history, a first malaria infection was favored to reduce the possibility of a recurrent *P*. *vivax* infection as the cause of the latest malaria episode.

### Apetina

Apetina, also known as Puleowime, a small Amerindian village with a non-mining indigenous population was the only other location in Suriname recording more than four *P*. *vivax* cases during the study period. Six from the nine cases diagnosed during the study period were included.

After the study period, no more indigenous malaria cases have been reported in any of the three aforementioned localities in Suriname.

### Sophie

Sophie, an illegal gold mining site in French Guiana just across the border river, was the site of infection resulting in the highest number of imported cases diagnosed in Suriname. Sophie is almost exclusively populated by a mobile migrant miner population. The isolates (n = 12) included from this region represented circulating strains in an area of active malaria transmission.

Selection of samples was restricted to samples diagnosed in Suriname and available in the National Malaria Gene Bank (NMGB). Whenever multiple samples were available within the same timeframe, preferential selection of samples from younger persons occurred, since younger persons may be more representative of a more recent genetic signature and have a lower risk of a previous malaria infection.

### Sampling & data collection

Forty-nine (n = 49) samples have been collected from participants with a single microscopy-confirmed *P*. *vivax* infection with Pelele Tepoe, Palumeu, Sophie or Apetina as presumed location of infection. No sex or age restrictions were applied.

All study samples were retrieved from the NMGB, a national repository with all microscopy-confirmed malaria positive cases stored as finger-prick dried blood spots (DBS) onto filter paper (3MM, Whatman Inc.)

Patient’s demographics and malaria history data was provided by the National Malaria Program.

### Size and sequence polymorphism analysis in *Pvmsp-1 F2* and *Pvmsp-3α* genes

Saponin/Chelex extracted DNA [16] from DBSs was amplified using master-nested PCR’s, targeting the *Pvmsp-1 F2* and *Pvmsp-3α* genes by previously described methods [13,14]. Size polymorphisms were detected by horizontal agarose electrophoresis in 1% agarose gels stained with ethidium bromide under UV illumination. Sequence polymorphisms were determined by RFLP using single enzyme digestions with each one of the following enzymes: *Alu*I, *Mnl*I (Thermo Fisher, Waltham, MA) for *Pvmsp-1 F2* and *Alu*I, *Hha*I (Thermo Fisher, Waltham, MA) for *Pvmsp-3α*. The resulting digested fragments were separated in 5% polyacrylamide gels by electrophoresis.

**Fig 2:**
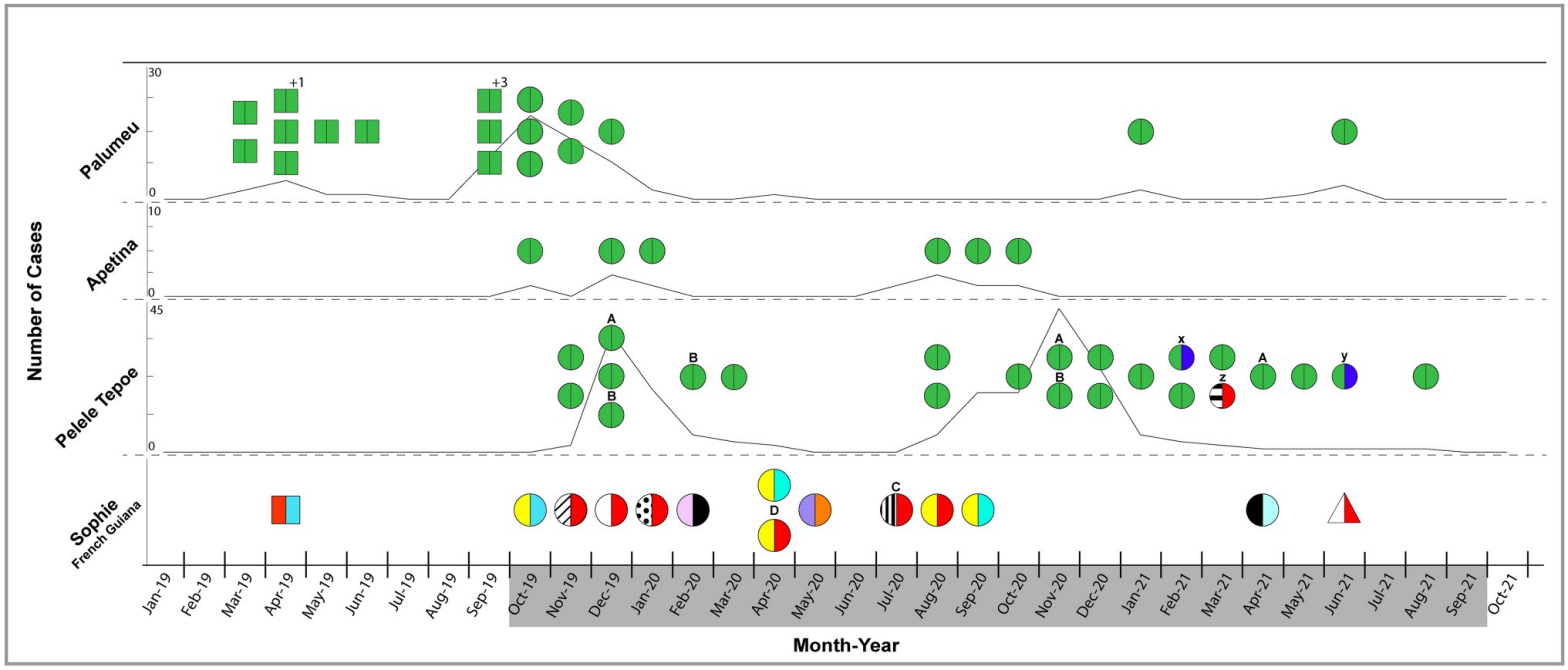
RFLP Profile Distribution for *Pvmsp-1 F2* and *Pvmsp-3*α per area. Trend lines for Palumeu, Apetina and Pelele Tepoe represent the total number of cases per region, diagnosed in Suriname in the period January 2019 to October 2021. The gray bar on the X-axis corresponds with the study period. Parasite isolates investigated within the study period are depicted as circles divided in two halves. Circle size in Sophie was increased just to achieve a better visual representation. Data from earlier molecular characterizations on parasite isolates outside the study period are symbolized by divided squares. Different allelic profiles for each of the genes *Pvmsp-1 F2* and *Pvmsp-3α* are linked to a different color or different pattern. Identical RFLP-profiles within a gene are presented with the same color. The color or pattern on the left side of each circle represents the corresponding *Pvmsp-1 F2* RFLP profile, while the color on the right side represents the corresponding *Pvmsp-3α* gene profile. Letters A and B mark two pregnant/breastfeeding participants from Pelele Tepoe with recurring malaria. Letters C and D denote two participants from Sophie, who experienced previous malaria episodes. Letters x, y and z denote participants from Pelele Tepoe with diverging allelic variants. An additional sample from Taluen, French Guiana collected in June 2021 is depicted as a triangle in the Sophie panel.

The RFLP patterns were translated into a six-digit code to facilitate the comparative analysis of isolates. The first three characters reflected the pattern for the *Pvmsp-1 F2* gene (size polymorphism, *Alu*I and *Mnl*I pattern), while the remaining three characters denoted the *Pvmsp-3α* gene profile (size polymorphism, *Alu*I and *Hha*I pattern). Infections displaying deviations in one or more of the four restriction patterns [different codes] were considered heterologous infections, while identical restriction profiles [identical code] were designated as homologous infections. Heterologous infections are represented in Fig 2, with a different color or different pattern, while homologous infections are presented with the same color.

### Statistical analysis

Fisher’s Exact Test (R version 4.2.2) was applied to compare genetic differences for each gene (*Pvmsp-1 F2* and *Pvmsp-3α*) among the sample sets from the different villages.

Lilliefors-corrected Kolmogorov-Smirnov test (R version 4.2.2) was used to test for uniform distribution of gene profiles.

The statistical significance level was set at p<0.05.

## Results

### Demographic data

The median age of the study participants (n = 49) was 21 years with a range from 1–80 years. The male to female ratio in the study sample was 1.13:1.

### Diversity of the Pvmsp-1 F2 and Pvmsp-3α gene

All DBS from field isolates could be successfully extracted and amplified for the *Pvmsp-1 F2* and *Pvmsp-3*α gene. *Pvmsp-1 F2* genotyping based on size polymorphisms rendered the two types A (1150 base pairs [bp]) and B (ca 1087 bp), corresponding to size variants described earlier [13]. Further classification of the *Pvmsp-1 F2* gene based on sequence polymorphisms yielded 7 and 8 different restriction profiles for each of the restriction enzymes *Alu*I and *Mnl*I respectively. The combination of size variants with both restriction patterns of *Pvmsp-1 F2* polymorphisms yielded 10 allelic types.

The PCR amplicons of the *Pvmsp*-*3α* gene revealed two discrete size variants Type A (1.9 kilobases [kb]) and Type B (1.5 kb), corresponding with the allele sizes, occurring worldwide. The most common allelic variant was type A, consistent with the global occurrence [17]. On the other hand, size variants type C (1.2 kb) and type D (0.3–0.5 kb) were not observed in this study cohort.

*Pvmsp-3α* sequence polymorphisms rendered 7 allelic types for *Alu*I and 7 allelic types for *Hha*I. The combination of both restriction patterns yielded 7 haplotypes and inclusion of size variants did not augment the results.

Genotyping based on size and sequence polymorphisms of both genes allowed us to distinguish 12 haplotypes among the 49 isolates.

### Determination of RFLP-genetic signatures in Palumeu, Apetina, Pelele Tepoe and Sophie

The results of the PCR-RFLP profile determination are visualized in Fig 2 as distribution of clonal lineages in time for each of the four investigated areas.

Parasite isolates are depicted as circles or squares divided in two colored halves, representing the *Pvmsp-1 F2* profile on the left side and the *Pvmsp-3α* profile on the right side. The total number of cases diagnosed per region in the presented period is shown as national trend lines for Palumeu, Apetina and Pelele Tepoe (data Ministry of Health Malaria Program Suriname [NMP]). No trend line was added for Sophie (French Guiana), since this study did not include malaria cases, diagnosed in French Guiana.

The outbreak and post-outbreak samples from Palumeu matched the variant Aa1-Aa1, which was earlier observed as single clonal lineage during the outbreak and designated as the “Palumeu haplotype” [12]. In addition, all six isolates collected from Apetina matched this “Palumeu haplotype”.

Moreover, even the majority (87%) of the investigated samples from the Pelele Tepoe outbreak were regarded as homologous infections also harboring this “Palumeu haplotype”.

In order to gain some insight in the evolution of genetic signatures with multiplication in the liver, paired isolates from two patients with recurring infections in Pelele Tepoe within the study period were included. As both patients were either pregnant or breastfeeding, relapses were suspected due to the non- use of Primaquine. The paired samples indeed harbored the “Palumeu haplotype” (Fig 2, patient A and B), despite a time interval of even 16 months between the first and third episode of patient A. (Fig 2).

Interestingly, the only three samples in Pelele Tepoe with diverging variants were collected from patients with a cross border (Brazil) travel history (Fig 2, patients x, y and z).

A homologous infection other than the “Palumeu haplotype” was demonstrated in a mother-child pair (Fig 2, patients x, y) and the third isolate harbored yet another variant. The deviating RFLP profiles did not match any other sample within this survey. To gain more insight in the origin of these RFLP-profiles, other cases diagnosed in Suriname in the period of the deviating RFLP patterns were compared and a supplemental sample, presumably originating from Taluen, French Guiana was also tested. Neither the two post-outbreak cases from Palumeu, nor the isolate from Sophie, nor the additional sample from Taluen (Fig 2, presented as triangle) matched the deviating RFLP patterns.

The PCR-RFLP analysis of the isolates from Sophie (n = 12) did not reveal the “Palumeu haplotype” and no less than 9 variants could be demonstrated (Fig 2).

The utilized PCR-RFLP displayed adequate discriminating capacity as the strains from Sophie were significantly different from the strains collected in Palumeu, Apetina and Pelele Tepoe (p<0.001), even if only the *Pvmsp-1 F2* gene (p<0.001) or just the *Pvmsp-3α* gene (p<0.001) would be considered.

It should be noted that *Pvmsp-1 F2* showed a higher degree of diversity than *Pvmsp-3α*.

Interestingly, samples from Sophie exhibited a non-uniform distribution for the *Pvmsp-1 F2* gene (p<0.001), with a high prevalence for one specific RFLP-profile and also for the *Pvmsp-3α* gene, two more common RFLP-profiles could be observed in the non-uniform distribution, although borderline significant (p = 0.046).

Complementary RFLP analysis was performed on paired isolates from previous episodes from two patients from Sophie (Fig 2, patient C and D), presumably also contracted in Sophie. The time interval between subsequent samples was 38 months for patient C and 33 months between the first and third episode of patient D.

The previous samples displayed novel allelic variants and all five isolates had diverging haplotypes.

Frequency distributions for individual restriction patterns were not calculated, due to inadequate numbers in each group.

## Discussion

Within this study cohort neither *Pvmsp-3α* variant type C, nor the rare size variant type D were detected, as could be expected with the low frequencies reported in South American isolates [18].

The utilization of size and sequence polymorphisms of the *Pvmsp-1 F2* gene for RFLP profiling seemed to have a superior differentiating capacity than genotyping of *Pvmsp-3α*, contrary to studies from Thailand, Papua New Guinea and in Indian strains, where a similar degree of diversity in *Pvmsp-1 F2* and *Pvmsp-3α* genes was reported [19]. However, the high frequency of occurrence of one single *Pvmsp-3α* variant in the samples originating from Sophie, an area with active malaria transmission and the highest degree of genetic diversity, could account for this finding.

The observed difference in genetic heterogeneity between the two investigated genes might be explained by variation in selective pressure for each gene, thus impacting the genetic differentiation in a different way than genetic drift alone [20].

However, population-specific polymorphisms linked to a geographic region of origin [4] seems more plausible, since all isolates were derived from migrants of Brazilian origin involved in illegal mining at Sophie and the fact that the strains not belonging to the clusters of identical patterns as observed in both *Pvmsp-1 F2* and *Pvmsp-3α* display diverging profiles.

Unfortunately, the limited number of samples from Sophie and the unreliable travel histories from miners, who usually provide mostly incomplete or incorrect histories because of the illegal nature of their activities, did not allow for further spatial clustering of allelic variants.

The increased mobility of illegal mobile miners and travel of villagers within Suriname and among communities in neighboring Brazil, Guyana, and French Guiana with significantly higher malaria transmission not only results in import cases, but also in introduced and locally acquired cases, due to our high density of malaria mosquitoes.

The single clonal lineage earlier reported for the Palumeu outbreak was also revealed in the post outbreak samples from Palumeu, suggesting either a relapse or a locally acquired secondary case, contracted through contact with person(s) from Pelele Tepoe.

The malaria cases observed in Apetina, the other indigenous village, also harbored the “Palumeu haplotype” implicating unregistered migration of people during an active malaria outbreak as cause for these individual cases.

The comparison of PCR/RFLP profiles of specimens throughout the lengthy outbreak in Pelele Tepoe, depicted as trend line in Fig 2, revealed a predominance of the single clonal type that matched a clonal lineage previously found in Palumeu.

These results corroborated the registered travel history, as no less than 60% of the first fifteen diagnosed cases, including the first diagnosed case in Pelele Tepoe had travelled to Palumeu.

RFLP results in conjunction with the epidemiological data, especially travel history obtained during testing or treating also pointed to undetected human movement during an active malaria outbreak as trigger for this subsequent Pelele Tepoe outbreak with locally acquired secondary cases, contracted via primary index case(s) imported via Palumeu.

It could be hypothesized that the prolongation of the outbreak with a second peak (Fig 2), despite several stringent measures as directly-observed treatment (DOT), organization of regular active case detection (ACD) and even two mass drug administration (MDA) events, reaching almost 90% of the residing population, was fueled by relapses from patients, who did not receive Primaquine because of a pregnancy, breastfeeding or glucose-6-phosphate dehydrogenase (G6PD) deficiency.

This hypothesis is substantiated by the homologous character of the RFLP profiles of the paired isolates from the breastfeeding/pregnant women with recurring infections, the absence of indications for drug resistance and the finding that 54% of recurrent malaria episode(s) in the Pelele Tepoe outbreak constituted of persons not receiving Primaquine.

However, it should be noted that even with correctly administered Primaquine treatment, relapse rates can range from 2.8–19.0% [21,22].

After the occurrence of the second peak in Pelele Tepoe, national precautions such as strict follow-up of pregnant women with biweekly microscopic monitoring till delivery and continued subsequent screening during breastfeeding were implemented in order to address reintroduction of malaria via possible relapses.

The introduction of the additional measures may have been instrumental in the success of fully containing the further spread of the “divergent” allelic variants, presumably imported from Brazil, as none of the other survey samples displayed the “divergent” haplotypes even though one of the returning travelers did not receive Primaquine, due to a pregnancy. The efficient suppression of the small-scale post-outbreak in Palumeu in May/June 2021, also involving two pregnant/breastfeeding women, provided further support for this notion.

The use of RFLP as molecular tool enabled reconstruction of the outbreak in Pelele Tepoe, elucidated the correlation between parasite strains circulating in the three indigenous villages and aided in the determination of the effectivity of measures implemented during the outbreak.

Our findings also implicated unregistered human movement and relapses as triggers for the reintroduction and resurgence of *P*. *vivax* malaria in the indigenous villages, thus requiring even more stringent malaria control and monitoring in all villages consisting of restricted travel during outbreaks and strict visitor-screening (including returning villagers), a strong awareness and monitoring system of symptoms in the community, especially for pregnant and breastfeeding women and early molecular characterization to guide the roll-out of interventions.

The unexpected phenomenon of a seemingly stable single-clone, throughout a period of more than two years with circulation in three indigenous villages, infecting and multiplicating in more than 250 persons, demonstrated significant inbreeding and infrequent or lack of recombination between heterologous parasite genotypes.

Even multiple relapses in different individuals and drug pressure exerted by several MDA events presumably did not add to the gene pool of the parasite, suggesting a limited *P*. *vivax* hypnozoite reservoir in Suriname, in contrast to the observations of maintained high levels of genetic diversity in other low-endemic areas as Sri Lanka [23].

The highly heterogeneous parasite population in the Sophie area in French Guiana (Fig 2) was in striking contrast to the near single clonal parasite population observed in Palumeu, Apetina and Pelele Tepoe, but consistent with the endemic malaria status in Sophie, maintained by the abundance of vectors and parasites and a dynamic mobile mining population. This finding was accentuated by the different allelic variants demonstrated in subsequent episodes of two patients (C and D) in Sophie, pointing to a high turnover of parasite genotypes, common in people living in high transmission areas, who can accumulate a pool of genetically diverse *P*. *vivax* hypnozoites through repeated *P*. *vivax* inoculations.

The findings in the indigenous villages on one hand and Sophie on the other hand also corroborated the common perception that declining transmission leads to a decreased genetic diversity, most likely due to diminished recombination between genetically distinct clones.

A more detailed spatial clustering of allelic variants in Sophie was prohibited, due to the limited number of samples from Sophie and the unreliable travel histories from miners.

Reduced genotyping accuracy as the common limitation of RFLP genotyping in areas of high- transmission with a high parasite density and high multiplicity of infection [24] was not an issue in this study, as the utilized PCR-RFLP was not only an effective tool in the outbreak investigations, but sufficient discriminating capacity could be demonstrated with the differentiation of several distinctive allelic variants from Sophie, even for the divergent strains in patients with recurring malaria in Sophie.

Notwithstanding the use of only two loci in the investigation, the findings from this study further substantiated the usefulness of PCR-RFLP as alternative for parasite genotyping analyses in hypo-endemic settings.

## Conclusions

The results from this study clearly demonstrated the differentiating capacity and therefore the usefulness of the utilized PCR-RFLP method with the *Pvmsp-1 F2* and *Pvmsp-3α* genes.

Therefore, we recommend the use of RFLP as molecular alternative for low endemic areas with restricted resource settings, especially for outbreak investigations, for monitoring of the spread or containment of circulating strains, for assessments of the degree of *P*. *vivax* genetic diversity and for identification of imported cases or potential foci.

Our findings highlighted the impact of human movement and relapses on the reintroduction and resurgence of *P*. *vivax* malaria in hypo-endemic settings and we strongly advocate for stringent and timely malaria prevention, control and monitoring measures during outbreaks, including early molecular characterization. Implementation of such measures will not only limit or prevent the reintroduction of malaria in near elimination areas, but will also restrict overall genetic diversity and impede the global spread of drug resistance.

## Supporting information

S1 Table Participant Demographics and RFLP profiles. (DOCX)

S2 Table Primer sequences/PCR programs for PCR-RFLP genotyping *P*. *vivax* parasites. (DOCX)
