# Supplementary material for: Advocating for PCR-RFLP as molecular tool within malaria programs in low endemic areas and low resource settings
Source: PLoS Negl Trop Dis. 2023 Nov 8;17(11):e0011747. doi: 10.1371/journal.pntd.0011747 (PMC10659184; doi:10.1371/journal.pntd.0011747)
Supplement: S1 Table — (DOCX) [file pntd.0011747.s001.docx]

**S1Table. Demographic Characteristics and RFLP-results for the *Pvmsp-1 F2* and *Pvmsp-3α* gene**

| **Sample ID^1^** | **Age** | **Sex** | **Collection date** | **RFLP Profile^2^** | **Symbol^3^** | **Geographic origin** |
| --- | --- | --- | --- | --- | --- | --- |
| #01 | 20 | M | 04-Nov-2019 | **Aa1-Aa1** |  | Pelele Tepoe (SUR) |
| #02 | 34 | V | 11-Nov-2019 | **Aa1-Aa1** |  | Pelele Tepoe (SUR) |
| #03 | 13 | M | 05-Dec-2019 | **Aa1-Aa1** |  | Pelele Tepoe (SUR) |
| #04**^A^** | 13 | V | 06-Dec-2019 | **Aa1-Aa1** |  | Pelele Tepoe (SUR) |
| #05**^B^** | 27 | V | 11-Dec-2019 | **Aa1-Aa1** |  | Pelele Tepoe (SUR) |
| #06**^B^** | 27 | V | 3-Feb-2020 | **Aa1-Aa1** |  | Pelele Tepoe (SUR) |
| #07 | 53 | V | 02-Mar-2020 | **Aa1-Aa1** |  | Pelele Tepoe (SUR) |
| #08 | 13 | M | 06-Aug-2020 | **Aa1-Aa1** |  | Pelele Tepoe (SUR) |
| #09 | 80 | V | 31-Aug-2020 | **Aa1-Aa1** |  | Pelele Tepoe (SUR) |
| #10 | 21 | M | 26-Oct-2020 | **Aa1-Aa1** |  | Pelele Tepoe (SUR) |
| #11**^A^** | 14 | V | 18-Nov-2020 | **Aa1-Aa1** |  | Pelele Tepoe (SUR) |
| #12**^B^** | 28 | V | 24-Nov-2020 | **Aa1-Aa1** |  | Pelele Tepoe (SUR) |
| #13 | 21 | M | 11-Dec-2020 | **Aa1-Aa1** |  | Pelele Tepoe (SUR) |
| #14 | 10 | M | 15-Dec-2020 | **Aa1-Aa1** |  | Pelele Tepoe (SUR) |
| #15 | 3 | M | 26-Jan-2021 | **Aa1-Aa1** |  | Pelele Tepoe (SUR) |
| #16 | 10 | V | 10-Feb-2021 | **Aa1-Aa1** |  | Pelele Tepoe (SUR) |
| #17 | 16 | V | 23-Feb-2021 | **Aa1-Ah10** |  | Pelele Tepoe (SUR) |
| #18 | 22 | M | 1-Mar-2021 | **Bc2-Bc5** |  | Pelele Tepoe (SUR) |
| #19 | 35 | M | 6-Mar-2021 | **Aa1-Aa1** |  | Pelele Tepoe (SUR) |
| #20**^A^** | 14 | V | 16-Apr-2021 | **Aa1-Aa1** |  | Pelele Tepoe (SUR) |
| #21 | 16 | V | 28-May-2021 | **Aa1-Aa1** |  | Pelele Tepoe (SUR) |
| #22 | 1 | M | 28-Jun-2021 | **Aa1-Ah10** |  | Pelele Tepoe (SUR) |
| #23 | 56 | M | 26-Aug-2021 | **Aa1-Aa1** |  | Pelele Tepoe (SUR) |
| #24 | 60 | V | 15-Oct-2019 | **Aa1-Aa1** |  | Apetina (SUR) |
| #25 | 36 | V | 9-Dec-2019 | **Aa1-Aa1** |  | Apetina (SUR) |
| #26 | 21 | M | 29-Jan-2020 | **Aa1-Aa1** |  | Apetina (SUR) |
| #27 | 15 | V | 25-Aug-2020 | **Aa1-Aa1** |  | Apetina (SUR) |
| #28 | 16 | M | 4-Sep-2020 | **Aa1-Aa1** |  | Apetina (SUR) |
| #29 | 20 | M | 28-Oct-2020 | **Aa1-Aa1** |  | Apetina (SUR) |
| #30 | 12 | M | 8-Oct-2019 | **Aa1-Aa1** |  | Palumeu (SUR) |
| #31 | 46 | M | 18-Oct-2019 | **Aa1-Aa1** |  | Palumeu (SUR) |
| #32 | 63 | V | 21-Oct-2019 | **Aa1-Aa1** |  | Palumeu (SUR) |
| #33 | 11 | M | 19-Nov-2019 | **Aa1-Aa1** |  | Palumeu (SUR) |
| #34 | 13 | M | 21-Nov-2019 | **Aa1-Aa1** |  | Palumeu (SUR) |
| #35 | 47 | M | 9-Dec-2019 | **Aa1-Aa1** |  | Palumeu (SUR) |
| #36 | 16 | M | 19-Jan-2021 | **Aa1-Aa1** |  | Palumeu (SUR) |
| #37 | 21 | V | 16-Jun-2021 | **Aa1-Aa1** |  | Palumeu (SUR) |
| #38 | 29 | V | 14-Oct-2019 | **Bs22-Ab2** |  | Sophie (F.G) |
| #39 | 36 | V | 1-Nov-2019 | **Aa20-Bc5** |  | Sophie (F.G) |
| #40 | 33 | V | 11-Dec-2019 | **Ak23-Bc5** |  | Sophie (F.G) |
| #41 | 38 | V | 16-Jan-2020 | **Bm2-Bc5** |  | Sophie (F.G) |
| #42 | 25 | M | 15-Feb-2020 | **Aa21-Af7** |  | Sophie (F.G) |
| #43**^D^** | 28 | M | 21-Apr-2020 | **Bs22-Bc5** |  | Sophie (F.G) |
| #44 | 1 | M | 27-Apr-2020 | **Bs22-Ab2** |  | Sophie (F.G) |
| #45 | 21 | M | 23-May-2020 | **Aa2-Ag8** |  | Sophie (F.G) |
| #46**^C^** | 48 | V | 31-Jul-2020 | **Bn24-Bc5** |  | Sophie (F.G) |
| #47 | 25 | M | 24-Aug-2020 | **Bs22-Bc5** |  | Sophie (F.G) |
| #48 | 33 | V | 1-Sep-2020 | **Bs22-Ab2** |  | Sophie (F.G) |
| #49 | 41 | M | 26-Apr-2021 | **Bt26-Aj11** |  | Sophie (F.G) |
| #50**^C^** | 45 | V | 17-May-2017 | **Bl20-Ab2** |  | Sophie (F.G) |
| #51**^D^** | 25 | M | 20-Jul-2017 | **Ak23-Ag8** |  | Sophie (FG) |
| #52**^D^** | 27 | M | 11-Dec-2018 | **Ao1-Ag9** |  | Sophie (FG) |
| #53 | 32 | V | 26-Apr-2019 | **Bf2-Ab2** |  | Sophie (FG) |
| #54 | 27 | V | 28-Jun-2021 | **Ak23-Bc5** |  | Taluen (FG) |

^1^Sample ID is depicted with consecutive arabic numerals; superscript letters are used as identifier for patients with recurrent infections.

^2^Allelic patterns are presented as a six-character code. Size type for each gene product is represented with letters (caps), while different restriction patterns are labeled with different lower-case letters and numbers. The order in numbers and lower-case letters is random. The first three characters reflect the pattern for the *Pvmsp-1 F2* gene (size polymorphism, *Alu*I and *Mnl*I pattern), while the remaining three characters denote the *Pvmsp-3α* gene profile (size polymorphism, *Alu*I and *Hha*I pattern).

**^3^**Parasite isolates investigated within the study period are depicted as circles divided in two halves.

Data from earlier molecular characterizations on parasite isolates outside the study period are symbolized by divided squares.

An additional sample from Taluen, French Guiana collected in June 2021 is depicted as a triangle in the Sophie panel.

Different allelic profiles for each of the genes *Pvmsp-1 F2* and *Pvmsp-3α* are linked to a different color or different pattern. Identical RFLP-profiles within a gene are presented with the same color. The color or pattern on the left side of each circle represents the corresponding *Pvmsp-1 F2* RFLP profile, while the color on the right side represents the corresponding *Pvmsp-3α* gene profile.

The symbols in the table correspond with the symbols in Figure 2.

Symbol size in Sophie and Taluen is increased just to achieve a better visual representation.

SUR: Suriname, F.G: French Guiana
