# Supplementary material for: Advocating for PCR-RFLP as molecular tool within malaria programs in low endemic areas and low resource settings
Source: PLoS Negl Trop Dis. 2023 Nov 8;17(11):e0011747. doi: 10.1371/journal.pntd.0011747 (PMC10659184; doi:10.1371/journal.pntd.0011747)
Supplement: S2 Table — (DOCX) [file pntd.0011747.s002.docx]

**S2 Table: Primer sequences/PCR programs for PCR-RFLP genotyping *P. vivax* parasites**

***Primer sequences***

| ***PvMSP1–F2 Region*** | | ***Sequence*** | ***Reference*** *[1]* |
| --- | --- | --- | --- |
| Master PCR | VMSP_1_ | GATGGAAAGCAACCGAAGAAGGGAAT |  |
|  | VMSP-N_1_ | AAAATCGAGAGCATGATCGCCACTGAGAAG |  |
| Nested PCR | VMSP-N_2_ | AGCTTGTACTTTCCATAGTGGTCCAG |  |
|  | VMSP-N_3_ | CATAGTGGTCCAGAGATACTTGTAGC |  |
| ***PvMSP3 Region*** | | ***Sequence*** | ***Reference*** *[2]* |
| Master PCR | MSP3-P1 | CAGCAGACACCATTTAAGG |  |
|  | MSP3-P2 | CCGTTTGTTGATTAGTTGC |  |
| Nested PCR | MSP3-N1 | GACCAGTGTGATACCATTAACC |  |
|  | MSP3-N2 | ATACTGGTTCTTCGTCTTCAGG |  |

***Details PCR Program***

|  | ***Gene PvMSP1–F2*** | | | |  | ***Gene PvMSP3*** | | | |
| --- | --- | --- | --- | --- | --- | --- | --- | --- | --- |
|  | ***Master*** | | ***Nested*** | |  | ***Master*** | | ***Nested*** | |
| Initial Denaturing: | 95°C | 5 min | 94°C | 5 min |  | 94°C | 3 min | 94°C | 3 min |
| Denaturing: | 94°C | 1 min | 94°C | 30 sec |  | 94°C | 30 sec | 94°C | 30 sec |
| Annealing: | **50°C** | 1 min | **55°C** | 45 sec |  | **49°C** | 1 min | **57°C** | 45 sec |
| Extension: | 72°C | 2 min | 72°C | 1 min |  | 68°C | 1:30 min | 68°C | 1:45 min |
| Number of cycles: | 35 | | 35 | |  | 35 | | 35 | |
| HotStart PCR | No | | No | |  | No | | Yes | |
| Final Extension: | 72°C | 5 min | 72°C | 5 min |  | 70°C | 5 min | 70°C | 5 min |

**Enzyme:** *Taq DNA Polymerase, recombinant (5 U/µL) Catalog number: 10342-178* [Invitrogen]

1. Imwong M, Pukrittayakamee S, Grüner AC, Rénia L, Letourneur F, Looareesuwan S, et al. Practical PCR genotyping protocols for *Plasmodium vivax* using Pvcs and Pvmsp1. Malar J. 2005;4:20. doi: 10.1186/1475-2875-4-20. PMID: 15854233; PMCID: PMC1131918.
2. Bruce MC**,** Galinski MR, Barnwell JW, Snounou G, Day KP. Polymorphism at the merozoite surface protein-3alpha locus of *Plasmodium vivax*: global and local diversity. Am J Trop Med Hyg. 1999;61(4):518-25. doi: 10.4269/ajtmh.1999.61.518. PMID: 10548283.
